# Enzymatic Synthesis of Glucose Monodecanoate in a Hydrophobic Deep Eutectic Solvent

**DOI:** 10.3390/ijms21124342

**Published:** 2020-06-18

**Authors:** Rebecca Hollenbach, Katrin Ochsenreither, Christoph Syldatk

**Affiliations:** Technical Biology, Institute of Process Engineering in Life Sciences II, Karlsruhe Institute of Technology, 76131 Karlsruhe, Germany; katrin.ochsenreither@kit.edu (K.O.); christoph.syldatk@kit.edu (C.S.)

**Keywords:** glycolipid, deep eutectic solvents, enzymatic synthesis, *Candida antarctica* lipase B, polarity

## Abstract

Environmentally friendly and biodegradable reaction media are an important part of a sustainable glycolipid production in the transition to green chemistry. Deep eutectic solvents (DESs) are an ecofriendly alternative to organic solvents. So far, only hydrophilic DESs were considered for enzymatic glycolipid synthesis. In this study, a hydrophobic DES consisting of (-)-menthol and decanoic acid is presented for the first time as an alternative to hydrophilic DES. The yields in the newly introduced hydrophobic DES are significantly higher than in hydrophilic DESs. Different reaction parameters were investigated to optimize the synthesis further. Twenty milligrams per milliliter iCalB and 0.5 M glucose resulted in the highest initial reaction velocity for the esterification reaction, while the highest initial reaction velocity was achieved with 1.5 M glucose in the transesterification reaction. The enzyme was proven to be reusable for at least five cycles without significant loss of activity.

## 1. Introduction

Surfactants are amphiphilic molecules that are applied in numerous industries and in personal care on a daily basis and a multimillion-ton scale per year [1,2,3,4]. The majority of surfactants are still based on fossil resources; however, due to environmental awareness there are also more ecofriendly alternatives, i.e., biosurfactants produced from renewables are getting more attention. Glycolipids, consisting of a sugar moiety acylated with an alkyl chain, are the biggest group within the biosurfactants. They are characterized by properties similar or even superior to their petrochemical counterparts while being biodegradable, non-toxic and skin-friendly [5,6,7]. 

Glycolipids can be synthesized by microbial fermentation, e.g., sophorose lipids and rhamnolipids, by chemical or enzymatic synthesis [8,9]. While microbial fermentation is limited to certain molecules, chemical and enzymatical synthesis can be used for tailor-made glycolipid synthesis with theoretically no restriction regarding sugar moiety and alkyl chain. Enzymatic synthesis additionally offers stereo- and regio-selectivity. For the linkage of saccharide and alkyl chain, a reaction solvent of low water activity is needed as reversed hydrolysis only occurs under conditions of reduced water activity [10,11,12]. Organic solvents are frequently used; however, the suitability of ionic liquids and deep eutectic solvents (DES) have also been studied. In contrast to organic solvents, DES and ionic liquids are non-volatile and non-flammable [13,14]. Furthermore, DES are reported to be biodegradable and non-toxic [15,16,17] while exhibiting a high dissolution power for many different materials, including drugs, proteins, salts, sugars and surfactants [18]. DES are applicable as solvent or catalyst in a wide range of organic reactions, e.g., addition, cyclization, condensation and multicomponent reactions, and improve the activity or selectivity of these reactions compared to organic solvents [19,20,21]. However, DES present a suitable solvent not only for organic reactions but also for biocatalysis. Lipases, glucosidases and proteases are among the enzymes that have been successfully used in DES to catalyze, for example, (trans-)esterification, Aldol and Henry reactions, as well as deglycosylation, dehalogenation, epoxide hydrolysis and oxidation reactions [14]. Initial reaction velocities as well as enzyme stability differ in various DES due to the varying hydrogen bond network depending on their constituents [18]. At the same time, a strong hydrogen bond network between DES and reaction substrates can lead to a limited availability of those for the reaction [22]. Therefore, it is necessary to select a DES suitable for substrates and enzymes. In some cases, a DES serves as solvent and as substrate for the enzymatic reaction [9]. 

However, the knowledge of glycolipid synthesis in DES is still limited. So far, only hydrophilic DES were used for glycolipid synthesis [9,23,24], although literature indicates that solvent polarity might have an impact on glycolipid synthesis. Enzyme stability is reduced in polar solvents due to stripping off hydration water from the enzyme [25]. Solvents of medium polarity present a compromise between enzyme stability and sugar solubility and showed, therefore, highest yields in organic solvents as well as in ionic liquids [26,27,28]. Furthermore, fatty acid availability is a limiting factor of glycolipid synthesis in hydrophilic DES [24].

Thus, the aim of this study was to investigate whether enzymatic synthesis of glycolipids is possible not only in hydrophilic DESs but also in a hydrophobic DES. For this purpose, a hydrophobic DES consisting of (-)-menthol and decanoic acid was used for the synthesis of glucose monodecanoate for the first time. Hydrophobic DES are a new class of solvents that were described in 2015 for the first time [29]. (-)-menthol: decanoic acid DES was chosen as this DES can serve as solvent and substrate simultaneously, and low water solubility was reported, which is beneficial for reversed hydrolysis reactions [30,31]. Furthermore, this DES was recently described to be suitable for an enzymatic reaction using *Candida rugosa* Lipase [32].

**Figure 1:**
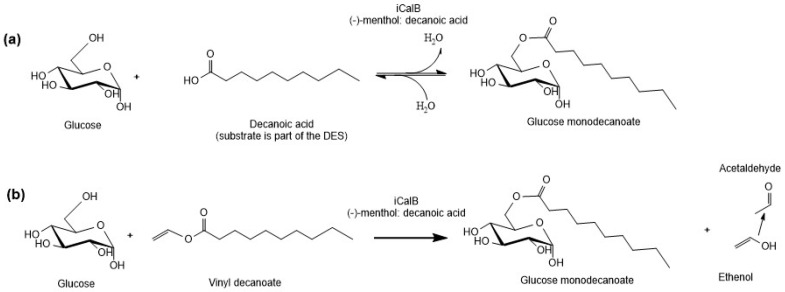
Reaction scheme of glucose monodecanoate synthesis. (**a**) Esterification reaction; (**b**) transesterification reaction. iCalB: immobilized *Candida antarctica* lipase B. DES: Deep eutectic solvents.

The glycolipid syntheses conducted in DESs have used mostly vinylated fatty acids as substrates, but no quantitative studies using free fatty acids in DESs have been conducted yet. However, the transesterification reaction introduces an additional reaction step as the fatty acids have to be vinylated prior to the reaction. Thus, the esterification reaction is preferred in terms of green chemistry. Therefore, esterification as well as transesterification were considered and compared in this study (Figure 1). Furthermore, the impact of the hydrophobic DESs on reaction rates was evaluated and several reaction parameters were examined in order to characterize the reaction in this novel reaction medium.

## 2. Results

The main purpose of this study was to examine the suitability of a hydrophobic (-)-menthol: decanoic acid DES as a medium for glycolipid synthesis and to compare the results with the synthesis in hydrophilic DES. Additionally, the influence of different reaction parameters was investigated to characterize the esterification as well as the transesterification reaction in this DES.

### 2.1. Reaction Time Course

The reaction time course was monitored with emphasis on the water released during esterification reaction, as water content is a crucial parameter for reversed hydrolysis. Within the transesterification reaction, water is not released. Although it might be interesting to determine water content for both reactions, water content measurements were only feasible for the reactions which did not contain vinyl decanoate as acetaldehyde interferes with the analysis. Therefore, water content can only be presented for the esterification reaction.

**Figure 2:**
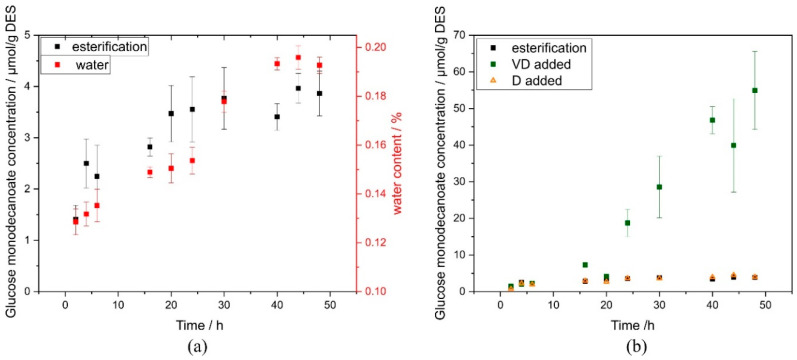
Reaction time course of glucose monodecanoate synthesis. (**a**) Glucose monodecanoate synthesis and water release during esterification reaction; (**b**) comparison of glucose monodecanoate synthesis in esterification, in esterification reaction with added decanoic acid (D added) and in transesterification reaction (VD added). D and VD were added after deep eutectic solvents (DES) production and before enzyme addition. D: decanoic acid; VD: vinyl decanoate.

Glucose monodecanoate concentration and water content were both rising in the first 24 h of reaction (Figure 2a). Subsequently, glucose monodecanoate production stagnated while water content increased further. 

In order to investigate whether the stagnation of product formation could be caused by a limited availability of the fatty acid in the DES, a further reaction set-up was carried out in which decanoic acid was supplemented in addition to the decanoic acid contained in the DES. The reaction progression remained unaltered when 0.5 M decanoic acid was added, causing no difference in comparison to the reaction without fatty acid addition (Figure 2b).

Transesterification reactions in which vinyl decanoate was applied additionally showed a lag phase over the first 16 h of reaction time. Due to this lag phase, the transesterification reaction and esterification reactions in which decanoic acid was used simultaneously for DES formation and as substrate did not differ in product formation rate within the first 6 h of reaction (Figure 2b). Subsequently, however, the product formation rates are significantly higher in transesterification reactions. Product yield after 24 h was 18.73 ± 3.73 μmol/g DES in contrast to 3.55 ± 0.63 μmol/g DES for esterification. At 48 h, the difference between both reaction set-ups was even higher, with 54.93 ± 10.66 μmol/g DES with added vinyl decanoate compared to 3.86 ± 0.43 μmol/g DES without. No product formation nor side product formation was observed in the negative controls (without addition of glucose). Chromatograms clearly indicate that (-)-menthol decanoate was not formed under the conditions applied within 120 h using iCalB as enzyme (Supplementary Materials Figure S1). 

### 2.2. External Mass Transfer

**Figure 3:**
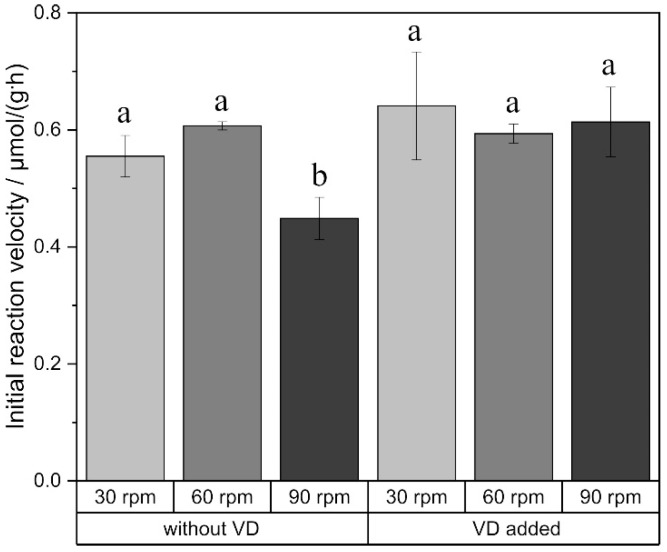
Initial reaction velocity in relation to the agitation rate. a, b indicate statistically significant differences. VD: vinyl decanoate.

The viscosity of the (-)-menthol: decanoic acid DES is 20.5 ± 0.05 mPa·s at 20 °C and 5.1 ± 0.09 mPa·s at 50 °C. The viscosity of water is 1.0 ± 0.01 mPa·s at 20 °C and 0.5 ± 0.01 mPa·s at 50 °C. Thus, mass transfer limitation is possible due to the higher viscosity of the DES compared to water. Hence, mixing is a crucial parameter. Therefore, the initial reaction velocity was investigated as a function of the agitation rate to evaluate external mass transfer. An increase in agitation rate increased the initial reaction velocity neither in the reaction with only free fatty acid nor in the reaction with added vinyl decanoate (Figure 3). 

### 2.3. Effect of Enzyme Concentration

**Figure 4:**
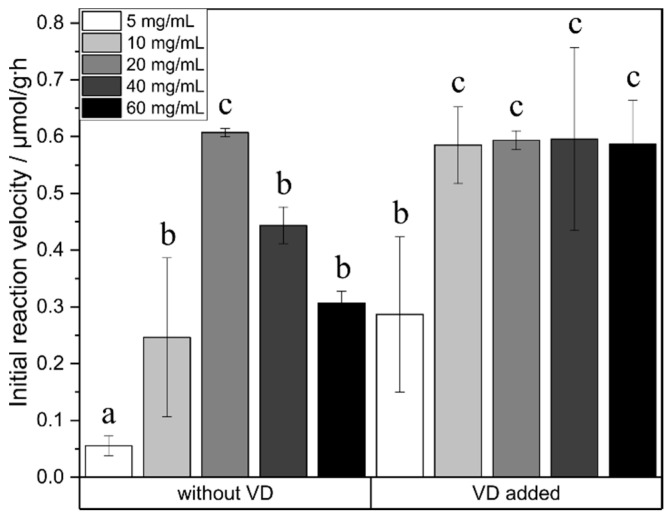
Impact of enzyme concentration on initial reaction velocity. a, b, c show statistically significant differences. VD: vinyl decanoate.

In order to find the best reaction conditions, enzyme concentration was evaluated. For the reaction without vinyl decanoate, a maximum was observed for the enzyme concentration (Figure 4). Initial reaction velocity increased with rising enzyme concentration up to 20 mg/mL. However, at higher enzyme concentrations, the initial reaction rate dropped. For the reactions with vinyl decanoate, this trend was not observed. Instead, enzyme concentrations from 10 mg/mL to 60 mg/mL resulted in the same initial reaction velocity as the standard reaction without vinyl decanoate at 20 mg/mL enzyme concentration. 

### 2.4. Effect of Initial Glucose Amount

**Figure 5:**
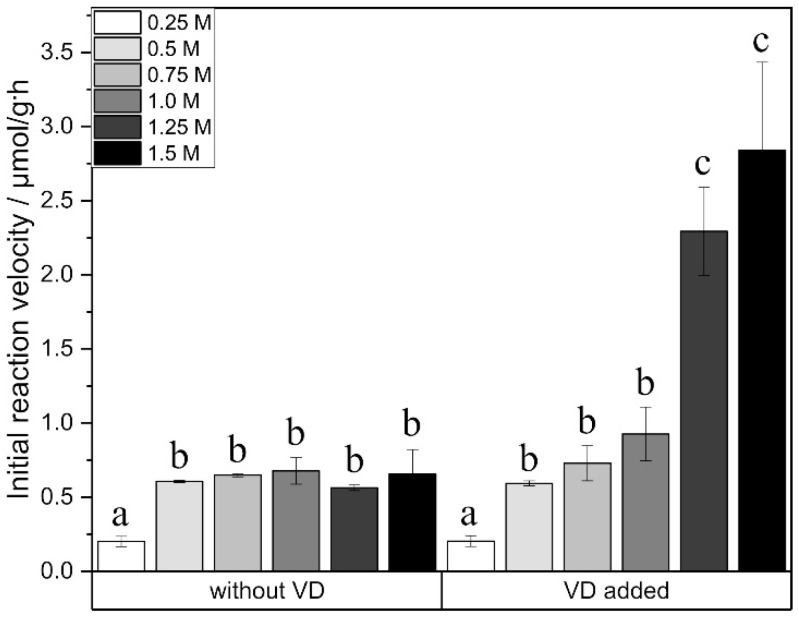
Initial reaction velocity in relation to different glucose amounts. a, b, c show statistically significant differences. VD: vinyl decanoate.

To evaluate suitable reaction parameters, the addition of glucose was also investigated. A glucose amount of 0.25 M instead of 0.5 M resulted in lower initial reaction velocity for both reactions, the one without vinyl decanoate as well as for the one with added vinyl decanoate (Figure 5), respectively. Further increase in glucose amount had no influence on the initial reaction velocity of the esterification reaction without vinyl decanoate. In contrast, the reaction rate of the synthesis with added vinyl decanoate was significantly enhanced by increasing sugar amounts. Increasing the glucose amount to 1.5 M improved the glucose monodecanoate yield significantly to 164.27 ± 9.98 μmol/g DES at a reaction time of 24 h.

### 2.5. Reusability of Enzyme 

The reusability of the enzyme is of interest especially for industrial applications as the catalyst costs are getting negligible with a rising number of reaction cycles. Therefore, we investigated the development of enzyme activity over several reaction cycles. In preliminary experiments, the lyophilization time of the washed enzyme was evaluated by measuring the water content. The water content of the fresh enzyme formulation was 1.55 ± 0.06%. After 24 h of lyophilization of the washed enzyme, the water content was 1.82 ± 0.10%. However, an extended lyophilization time (up to 120 h) did not reduce the water content further.

**Figure 6:**
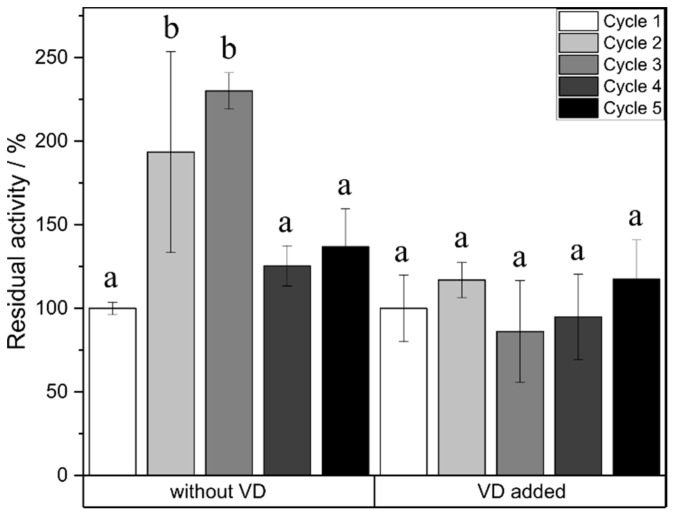
Residual activity of the enzyme in relation to the reaction cycle. Yield of reaction cycle 1 after 24 h was set to 100%. a, b show statistically significant differences. VD: vinyl decanoate.

For the esterification reaction, no loss of activity was observed over five reaction cycles (Figure 6). Cycles 2 and 3 showed a significantly higher yield after 24 h of reaction compared to cycle 1. The relative activity of the enzyme remained the same over five cycles for the reaction with added vinyl decanoate.

## 3. Discussion

In this study, it was shown for the first time that a hydrophobic DES containing (-)-menthol and decanoic acid is suitable for enzymatic glycolipid synthesis. Remarkably, glucose monodecanoate yields in the investigated hydrophobic (-)-menthol: decanoic acid DES are 20 to 1000 times higher than those reported by Hollenbach et al., 2020, in hydrophilic DES (Table 1) [24]. The increase in productivity is likely caused by the difference in solvent polarity as solvent polarity is already reported to have an effect on glycolipid synthesis in organic solvents [26,27]. In ionic liquids, it is also reported that medium polarity is most appropriate to dissolve sugar as well as fatty acids [28]. The solvatochromic π* is a measure of polarizability and dipolarity of solvents. The π* value is much lower for the (-)-menthol: decanoic acid DES (0.35) than for hydrophilic DES containing choline chloride and urea (1.192) or choline chloride and glucose (1.161) [31,33]. Therefore, polarity can be assumed as a major parameter in glycolipid synthesis in DESs. 

The hydrophobic and hydrophilic DES differ not only in their polarity but also in their viscosity. The viscosity of the hydrophobic DES is 10–30 times lower than those reported for hydrophilic DES [24]. However, external mass transfer limitation can be excluded by sufficient mixing. Thus, viscosity seems to be only of minor importance in glycolipid synthesis in DES. For downstream processing, however, the lower viscosity of the (-)-menthol: decanoic acid DES compared to the hydrophilic DES might be beneficial. The different strengths and natures of the hydrogen bonding network in the different DESs contribute to their different viscosities, as well as likely to their different performances. The hydrogen bond acidity α and the hydrogen bond basicity β are both lower for the presented hydrophobic DES than for the hydrophilic ones [31,33]. 

Negative controls without addition of glucose were performed to exclude the formation of (-)-menthol decanoate as this reaction was reported in (-)-menthol: fatty acid DES with *Candida rugosa* lipase [32]. No product formation was detected in these negative controls. iCalB prefers primary hydroxy groups [34,35,36]. Menthol has a secondary hydroxy group with additional steric hindrance due to an adjacent isopropyl group.

Water content is reported to be a crucial parameter in reversed hydrolysis [11,12,33]. Water is released during esterification reaction (Figure 1a). Since water is consumed again in hydrolysis, the reaction equilibrium is shifted to the reactants’ side once a certain water content is reached [11,12,37,38]. During the esterification reaction, water content raised from 0.13% to 0.19% until stagnation as water is formed within the reaction.

Another possible limitation of the reaction might be bare availability of decanoic acid for the reaction since decanoic acid acts simultaneously as hydrogen bond donor in the DES. Strong associations between substrates and the hydrogen bond network of the DES are reported that cause a low availability of substrates [22]. However, limited availability of decanoic acid in the DES for the enzymatic reaction was excluded by addition of free fatty acid to the reaction, which caused no difference in the pattern of the reaction. Therefore, the increasing water content is most likely the limiting factor of the reaction with exclusively free fatty acid, leading to an equilibrium between synthesis and hydrolysis of glucose monodecanoate.

The transesterification reaction using vinyl decanoate as additional substrate resulted in 6 times higher yields than the esterification even though there is no difference in the reaction time course during the first 6 h. During transesterification reaction, water is not formed, which might shift the equilibrium to hydrolysis. Instead, ethenol is formed that tautomerizes to its corresponding aldehyde, acetaldehyde, and evaporates (Figure 1b). Thus, the reaction is shifted towards the product side. This is likely the reason why the yield of the transesterification reaction is higher than that of the esterification reaction.

External mass transfer limitation can be excluded under the conditions used as the initial reaction velocity remains unaltered at increased reaction rates. The maximum in the initial reaction velocity depending on the agitation rate might be due to a loss of enzyme activity at higher shaking rates. An optimum in agitation rate was also observed by Zhao et al., 2011, in mono- and diglyceride synthesis [39]. The absence of that maximum for the synthesis with added vinyl decanoate is most likely because half the amount of enzyme leads to the same initial reaction velocity as 20 mg enzyme/mL for the transesterification reaction. The investigations of the enzyme concentration showed an increased initial reaction velocity with increasing enzyme concentration up to 10 mg/mL. At higher concentrations, a stagnation of the initial reaction velocity was observed due to saturation. However, an optimum in enzyme concentration was determined for the esterification reaction. Due to the water formation within this reaction, an increased enzyme concentration might lead to a higher velocity of the hydrolysis reaction, which causes lower product yields. Nevertheless, the higher enzyme concentration also led to an increased initial water concentration due to the water within the matrix of the enzyme formulation. The initial water content at an enzyme concentration of 60 mg/mL is in the range of the water content at a steady state of the esterification reaction when using an enzyme concentration of 20 mg/mL. Therefore, the inhibitory effect could also be ascribed to the water content. Similar findings were already observed in glucose ester synthesis in organic solvents [40].

The initial reaction velocity increased with increasing glucose amounts although glucose had not been completely dissolved in any of the applied concentrations. The observed results might be due to a faster glucose dissolution at higher glucose amounts. This effect could only be observed for the transesterification reaction; for the esterification reaction, though, a stagnation occurred at 100 mg/mL glucose. Transesterification might be faster than esterification because of its thermodynamic advantage. Therefore, the amount of glucose has a stronger influence on transesterification than on esterification.

The investigations on the reusability of the enzyme over five reaction cycles showed no significant loss of activity for the esterification reaction nor for the transesterification reaction. Pre-incubation of iCalB in the hydrophilic DES, choline chloride: glycerol and choline chloride: urea was reported to cause a significant loss of activity of 70% and 38% [41]. The higher stability of iCalB in the (-)-menthol: decanoic acid DES might be due to the lower polarity of the latter. Solvent polarity is reported to have a relation to enzyme activity and stability as more polar solvents strip off hydration water from the enzyme [25,42].

## 4. Materials and Methods 

### 4.1. Materials

Glucose, (-)-menthol and all solvents (HPLC grade) were acquired from Carl-Roth (Karlsruhe, Germany). Lipase B from *Candida antarctica*, immobilized on acrylic resin (iCalB), was purchased from Strem Chemicals (Strem chemicals Europe, Germany). Vinyl decanoate and decanoic acid were acquired from Tokyo Chemical Industry Co., Ltd. (TCI Europe, Belgium). 6-Decanoyl-d-glucose was purchased from Sohena (Tübingen, Germany). 

### 4.2. Viscosity Measurements

Viscosity measurements were performed using a Physica MCR 101 viscosimeter (Anton Paar, Ostfildern, Germany) with double gap geometry (DG26.7) at temperatures of 20 °C and 50 °C. Measurements were conducted at shear rates of 2–100 s^−1^.

### 4.3. Water Content Analysis

The water content was determined by Karl-Frischer titration using a TitroLine 7500 KF trace from SI Analytics (Mainz, Germany) at 20 °C using Aquastar CombiCoulomat fritless (Merck Millipre, Darmstadt, Germany) as an analyte. A water standard of Merck Millipore (Darmstadt, Germany) was used to test the titrator before the measurements. 

### 4.4. Glycolipid Synthesis

Decanoic acid and (-)-menthol were mixed in a molar ratio of 1:1 in a glass bottle at 80 °C for 1 h until a homogenous liquid was obtained. For all reactions, 1 mL DES was transferred to 5 mL reaction tubes, and glucose (final concentration 0.5 M) was added. For the transesterification reactions, 0.5 M vinyl decanoate was supplemented additionally. For the reaction with additional decanoic acid, decanoic acid (final concentration 0.5 M) was supplemented after the DES production. Finally, 20 mg/mL iCalB were added to start the reaction. The tubes were mixed in a rotator with a vortex mixer (program U2) from neoLab (Heidelberg, Germany) at 60 rpm and 50 °C. Samples for HPLC measurements were taken at distinct timepoints, diluted with ethyl acetate and analyzed by HPLC-ELSD.

### 4.5. Initial Reaction Velocity

To determine the initial reaction rates, glycolipid synthesis was carried out as described above (50 °C, 90 rpm), and samples for HPLC analysis were taken after 4 h.

### 4.6. Influence of Enzyme Concentration

In order to evaluate the impact of enzyme amount on product formation and initial reaction rates, the following enzyme concentrations were investigated without changing any other parameters: 5 mg/mL, 10 mg/mL, 20 mg/mL, 40 mg/mL and 60 mg/mL iCalB.

### 4.7. Optimization of Glucose Amount

To examine the effect of the added glucose amount on initial reaction rates, an enzyme concentration of 20 mg/mL was applied while keeping the other reaction parameters unchanged. The following final glucose concentrations were tested: 0.25 M, 0.5 M, 0.75 M, 1.0 M, 1.25 M and 1.5 M. After 4 h, samples were taken to determine the initial reaction velocity.

### 4.8. Reusability of Enzyme

Reusability of iCalB was tested for esterification as well as transesterification reactions. For esterification, 0.5 M glucose, 20 mg/mL iCalB and 1 mL DES were applied; for transesterification, 1.5 M glucose, 0.5 M vinyl decanoate and 20 mg/mL iCalB were added to 1 mL DES. After 24 h synthesis, the mixture was filtered using a Büchner funnel. The enzyme was washed three times with ethyl acetate to get rid of remaining DES and three times afterward with distilled water to get rid of remaining sugar. Then the enzyme was freeze-dried with a DW-10N freeze drier from Drawell (Shanghai, China) for 48 h. Afterward, the dried enzyme was reused for another synthesis. The conversion in the first cycle was set to 100% to calculate the residual activity of the enzyme in the following cycles.

### 4.9. HPLC-ELSD-Analysis

Samples were analyzed by reversed-phase HPLC according to the method described by Hollenbach et al., 2020 [24]. The HPLC system was equipped with a Kinetex EVO C18 column (2.6 μm, 250 × 4.6 mm) from Phenomenex (Aschaffenburg, Germany) with an accompanying guard column (4 × 3.0 mm ID) of the same phase using an Agilent (Waldbronn, Germany)1260 series liquid chromatograph equipped with a quaternary pump, an autosampler and a column oven. Analytes were detected using an evaporative light scattering detector from BÜCHI Labortechnik (Essen, Germany). The retention times were 2.1 min for glucose and 2.7 min for glucose monodecanoate.

### 4.10. Statistical Analysis

Results are given as mean ± standard deviation (*n* = 3). Statistical data analysis was performed by two-way ANOVA and Tukey test. For this, the software OriginPro 9.6 (version 2019; OriginLab, Nothampton, MA, USA) was used. Results were considered as significant if *p*-value was < 0.05.

## 5. Conclusions

The aim of this study was to investigate the applicability of a hydrophobic (-)-menthol: decanoic acid DES for glycolipid synthesis. The glucose monodecanoate yields were significantly improved by using the newly introduced DES compared to the hydrophilic DES used so far. The polarity of the used solvent was identified as crucial for glycolipid productivity. Furthermore, the reaction was also possible with free fatty acids instead of the thermodynamically preferred reaction with vinylated fatty acids. Therefore, the additional reaction step generating vinylated fatty acids can be omitted, as well as the highly volatile side product acetaldehyde. Moreover, the enzyme showed high stability and reusability in (-)-menthol: decanoic acid DES without loss of activity for at least five reaction cycles.

## Figures and Tables

**Table 1:** Comparison of glucose monodecanoate yields in (-)-menthol: decanoic acid DES and in hydrophilic DES.

Different DES	Glucose Monodecanoate Yield (24 h)
Choline chloride: urea DES with VD	0.15 μmol/g DES (0.03%) [24]
(-)-menthol: decanoic acid DES	3.55 μmol/g DES (0.71%)
(-)-menthol: decanoic acid DES with VD (0.5 M glucose)	18.73 μmol/g DES (3.75%)
(-)-menthol: decanoic acid DES with VD (1.5 M glucose)	164.27 μmol/g DES (10.95%)

VD: vinyl decanoate; yields [%] were calculated based on the glucose concentration, with a theoretical yield of c (glucose) = c (glucose monodecanoate) = 100%.

## Supplementary Materials

Supplementary materials can be found at https://www.mdpi.com/1422-0067/21/12/4342/s1.

## Abbreviations

DES	Deep eutectic solvent
D	Decanoic
VD	Vinyl decanoate
