# Supplementary material for: Enzymatic Synthesis of Glucose Monodecanoate in a Hydrophobic Deep Eutectic Solvent
Source: Int J Mol Sci. 2020 Jun 18;21(12):4342. doi: 10.3390/ijms21124342 (PMC7352255; doi:10.3390/ijms21124342)
Supplement: Supplementary file 1 [file ijms-21-04342-s001.pdf]

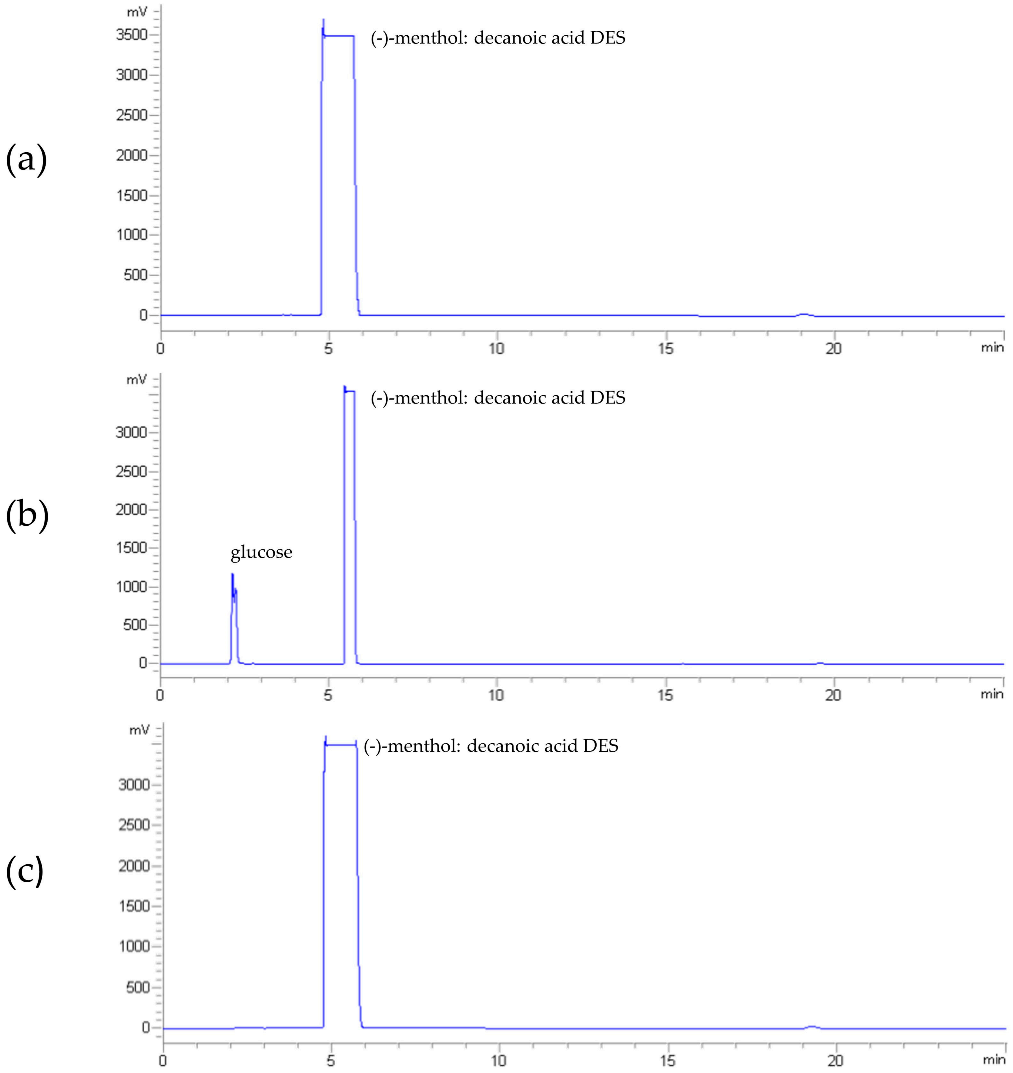

Supplementary Figure S1. HPLC-ELSD-Chromatograms of pure DES and negative controls of the synthesis. Chromatograms clearly indicate that no product nor side product formation occurs in the negative controls. a: pure (-)-menthol: decanoic acid DES diluted with ethyl acetate; b: negative control without addition of enzyme at 120 h reaction time; c: negative control without addition of glucose at 120 h reaction time.
